# Supplementary material for: Modeling glioblastoma heterogeneity as a dynamic network of cell states
Source: Mol Syst Biol. 2021 Sep 16;17(9):e10105. doi: 10.15252/msb.202010105 (PMC8444284; doi:10.15252/msb.202010105)
Supplement: Supplementary file 6 — Source Data for Figure 5 [file MSB-17-e10105-s004.zip › Figure5A_sourcedata/GSEA_3071/nosigdb_stateIV.GseaPreranked.1622795164626/NB_KI_CLUSTER4.html]

Details for gene set NB\_KI\_CLUSTER4[GSEA]

|  || Dataset | state53071 |
| Phenotype | NoPhenotypeAvailable |
| Upregulated in class | na\_pos |
| GeneSet | NB\_KI\_CLUSTER4 |
| Enrichment Score (ES) | 0.57593864 |
| Normalized Enrichment Score (NES) | 2.8856418 |
| Nominal p-value | 0.0 |
| FDR q-value | 0.0 |
| FWER p-Value | 0.0 |
Table: GSEA Results Summary

  

Fig 1: Enrichment plot: NB\_KI\_CLUSTER4      
 Profile of the Running ES Score & Positions of GeneSet Members on the Rank Ordered List

  

| PROBE | GENE SYMBOL | GENE\_TITLE | RANK IN GENE LIST | RANK METRIC SCORE | RUNNING ES | CORE ENRICHMENT || 1 | FXYD6 |  |  | 19 | 2.252 | 0.0363 | Yes |
| 2 | GNAS |  |  | 31 | 2.010 | 0.0698 | Yes |
| 3 | CADM1 |  |  | 38 | 1.965 | 0.1035 | Yes |
| 4 | SYT11 |  |  | 67 | 1.740 | 0.1290 | Yes |
| 5 | TUBB2B |  |  | 69 | 1.731 | 0.1595 | Yes |
| 6 | CKB |  |  | 72 | 1.711 | 0.1895 | Yes |
| 7 | DPYSL3 |  |  | 75 | 1.691 | 0.2191 | Yes |
| 8 | RIC3 |  |  | 115 | 1.495 | 0.2381 | Yes |
| 9 | CXADR |  |  | 139 | 1.402 | 0.2586 | Yes |
| 10 | TUBA1A |  |  | 165 | 1.337 | 0.2775 | Yes |
| 11 | CDK6 |  |  | 178 | 1.299 | 0.2983 | Yes |
| 12 | MYEF2 |  |  | 225 | 1.196 | 0.3107 | Yes |
| 13 | ATP6V0E2 |  |  | 236 | 1.179 | 0.3297 | Yes |
| 14 | KIF21A |  |  | 239 | 1.171 | 0.3500 | Yes |
| 15 | RBP1 |  |  | 254 | 1.136 | 0.3675 | Yes |
| 16 | GTF2I |  |  | 329 | 0.990 | 0.3709 | Yes |
| 17 | ATP1B1 |  |  | 359 | 0.961 | 0.3824 | Yes |
| 18 | RUFY3 |  |  | 376 | 0.929 | 0.3958 | Yes |
| 19 | SOX4 |  |  | 377 | 0.928 | 0.4122 | Yes |
| 20 | ATCAY |  |  | 397 | 0.910 | 0.4247 | Yes |
| 21 | MARCKSL1 |  |  | 417 | 0.888 | 0.4368 | Yes |
| 22 | MDK |  |  | 495 | 0.819 | 0.4366 | Yes |
| 23 | ZNF704 |  |  | 498 | 0.818 | 0.4507 | Yes |
| 24 | PEBP1 |  |  | 516 | 0.802 | 0.4617 | Yes |
| 25 | MYL6B |  |  | 529 | 0.786 | 0.4734 | Yes |
| 26 | H3F3A |  |  | 532 | 0.783 | 0.4869 | Yes |
| 27 | PTMS |  |  | 549 | 0.763 | 0.4973 | Yes |
| 28 | TRIM2 |  |  | 577 | 0.741 | 0.5053 | Yes |
| 29 | RNF165 |  |  | 606 | 0.725 | 0.5128 | Yes |
| 30 | GLCCI1 |  |  | 611 | 0.719 | 0.5248 | Yes |
| 31 | GDAP1 |  |  | 617 | 0.715 | 0.5365 | Yes |
| 32 | STMN3 |  |  | 643 | 0.697 | 0.5441 | Yes |
| 33 | PHF14 |  |  | 662 | 0.681 | 0.5527 | Yes |
| 34 | NREP |  |  | 701 | 0.660 | 0.5571 | Yes |
| 35 | BEX3 |  |  | 707 | 0.655 | 0.5678 | Yes |
| 36 | BEX2 |  |  | 725 | 0.645 | 0.5759 | Yes |
| 37 | TCAF1 |  |  | 954 | 0.541 | 0.5419 | No |
| 38 | SYP |  |  | 955 | 0.541 | 0.5515 | No |
| 39 | GDI1 |  |  | 1002 | 0.521 | 0.5519 | No |
| 40 | FSCN1 |  |  | 1003 | 0.521 | 0.5611 | No |
| 41 | APLP1 |  |  | 1035 | 0.510 | 0.5642 | No |
| 42 | NELFCD |  |  | 1053 | 0.504 | 0.5699 | No |
| 43 | VAMP2 |  |  | 1092 | 0.494 | 0.5714 | No |
| 44 | TSPAN7 |  |  | 1181 | 0.471 | 0.5629 | No |
| 45 | ATAT1 |  |  | 1318 | 0.436 | 0.5446 | No |
| 46 | ATP6V0A1 |  |  | 1320 | 0.436 | 0.5521 | No |
| 47 | TUBB2A |  |  | 1373 | 0.422 | 0.5497 | No |
| 48 | CALM1 |  |  | 1409 | 0.414 | 0.5503 | No |
| 49 | CIRBP |  |  | 1436 | 0.406 | 0.5525 | No |
| 50 | TERF2IP |  |  | 1449 | 0.402 | 0.5574 | No |
| 51 | CHGB |  |  | 1466 | 0.398 | 0.5614 | No |
| 52 | RAMP2 |  |  | 1537 | 0.381 | 0.5547 | No |
| 53 | SNRPN |  |  | 1634 | 0.362 | 0.5428 | No |
| 54 | ZC3H13 |  |  | 1647 | 0.359 | 0.5468 | No |
| 55 | MTSS1 |  |  | 1708 | 0.348 | 0.5415 | No |
| 56 | OCIAD2 |  |  | 1906 | 0.313 | 0.5093 | No |
| 57 | MLLT11 |  |  | 1932 | 0.308 | 0.5100 | No |
| 58 | LUC7L3 |  |  | 2124 | 0.280 | 0.4784 | No |
| 59 | MCRIP1 |  |  | 2209 | 0.267 | 0.4670 | No |
| 60 | PRKAR2B |  |  | 2297 | 0.256 | 0.4549 | No |
| 61 | GATA3 |  |  | 3134 | -0.351 | 0.3010 | No |
| 62 | CYB561 |  |  | 3229 | -0.365 | 0.2895 | No |
| 63 | NME1 |  |  | 3548 | -0.419 | 0.2360 | No |
| 64 | HSP90AB1 |  |  | 3618 | -0.432 | 0.2304 | No |
| 65 | CALM2 |  |  | 3909 | -0.489 | 0.1836 | No |
| 66 | YWHAH |  |  | 4032 | -0.521 | 0.1694 | No |
| 67 | TUBB |  |  | 4083 | -0.534 | 0.1693 | No |
| 68 | MIAT |  |  | 4622 | -0.748 | 0.0795 | No |
| 69 | SLC38A1 |  |  | 4971 | -1.043 | 0.0314 | No |
| 70 | BASP1 |  |  | 5203 | -1.665 | 0.0167 | No |
Table: GSEA details [plain text format]

  

Fig 2: NB\_KI\_CLUSTER4: Random ES distribution      
 Gene set null distribution of ES for **NB\_KI\_CLUSTER4**
